# Inter-relationships between changes in stress, mindfulness, and dynamic functional connectivity in response to a social stressor

**DOI:** 10.1038/s41598-022-06342-0

**Published:** 2022-02-14

**Authors:** James Teng, Stijn A. A. Massar, Julian Lim

**Affiliations:** 1 Centre for Sleep and Cognition, Yong Loo Lin School of Medicine, National University of Singapore, Tahir Foundation Building, MD1, Level 13 South, 12 Science Drive 2, Singapore, 117549 Singapore; 2 Department of Psychology, National University of Singapore, Singapore, Singapore

**Keywords:** Stress and resilience, Cognitive neuroscience, Psychology

## Abstract

We conducted a study to understand how dynamic functional brain connectivity contributes to the moderating effect of trait mindfulness on the stress response. 40 male participants provided subjective reports of stress, cortisol assays, and functional MRI before and after undergoing a social stressor. Self-reported trait mindfulness was also collected. Experiencing stress led to significant decreases in the prevalence of a connectivity state previously associated with mindfulness, but no changes in two connectivity states with prior links to arousal. Connectivity did not return to baseline 30 min after stress. Higher trait mindfulness was associated with attenuated affective and neuroendocrine stress response, and smaller decreases in the mindfulness-related connectivity state. In contrast, we found no association between affective response and functional connectivity. Taken together, these data allow us to construct a preliminary brain-behaviour model of how mindfulness dampens stress reactivity and demonstrate the utility of time-varying functional connectivity in understanding psychological state changes.

## Introduction

**Figure 1:**
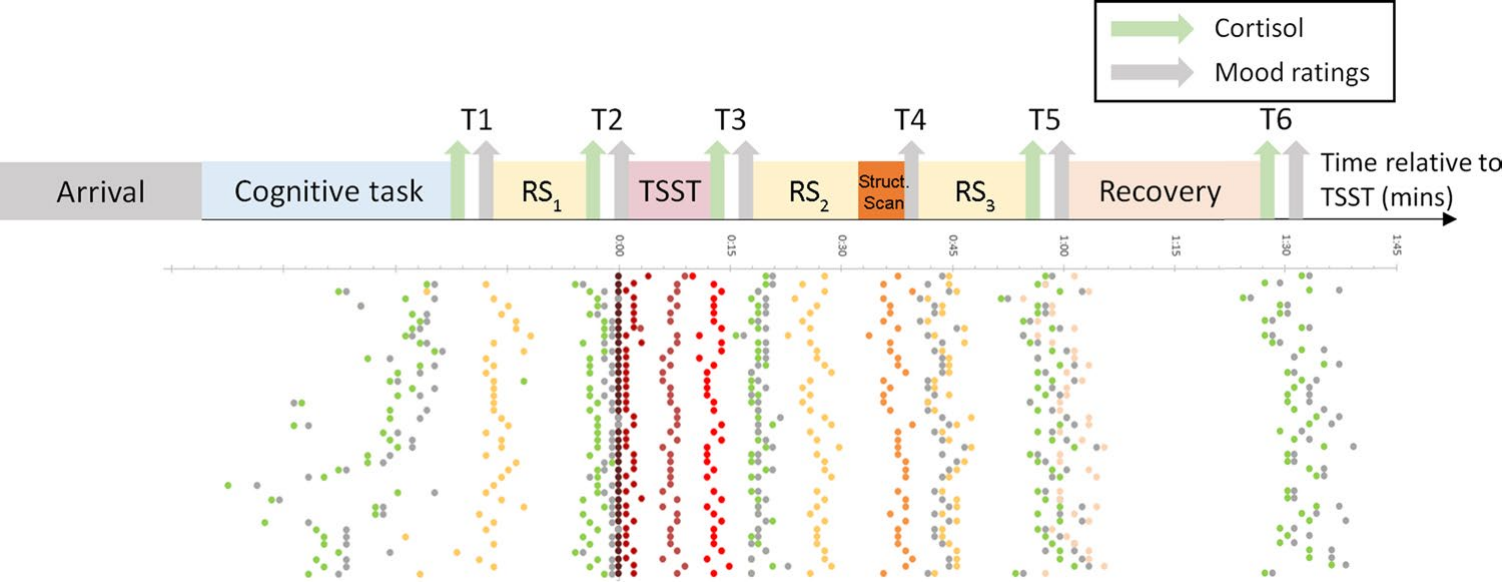
Schematic diagram of experimental protocol. Cortisol and subjective mood ratings were collected at 6 time points, and the main section of the protocol consisted of 3 resting-state fMRI scans, with administration of the Trier Social Stress Test (TSST) between scans 1 and 2. Each row of dots represents a single participant, and the time that they began each stage of the procedure relative to T1.

Facing a stressor triggers a complex cascade of physiological and psychological reactions that prepares the body to respond to physical threat. In social settings however, the effects of these reactions are largely undesirable, for example, having to deal with anxious thoughts and a racing heart while giving an important presentation. Using mindfulness—the practice of focusing one’s attention on the present moment while maintaining a non-judgmental and non-reactive stance to the experiences in it—is an effective strategy for coping with and dampening the effects of stress in such situations. While the psychological and biological changes associated with mindfulness and stress have been well studied separately, a model incorporating the mind, brain, and endocrine responses to their interaction has yet to be constructed. Here, we make a first attempt to connect some of these disparate pieces using cortisol and functional MRI connectivity changes resulting from the Trier Social Stress Test (TSST)^1^, a widely used laboratory procedure to induce stress (Fig. 1).

Undergoing the TSST causes transient but large increases in perceived stress, arousal, and cortisol release^2^. In addition, there is a growing body of evidence that acute stress also induces changes in resting-state functional connectivity. Exposure to social stress has been linked to increased connectivity between the default mode network and the amygdala^3–5^, and more generally to the salience network, while connectivity within the default mode network itself is reduced^6^. Other studies have found increased connectivity within the salience network (including amygdala, insula, and dorsal ACC)^7^, and between salience network and other areas (i.e. dlPFC)^6^. Furthermore, recent work has revealed that undergoing stress induction increases activity in hippocampal subregions, which predicts subsequent increases in brain areas related to emotion processing such as the insula^8^. While earlier studies have often focused on connectivity in a few seed regions, other studies using whole brain analysis have revealed a wider range of connectivity changes (both increased and decreased) due to acute stress^9–12^. These changes indicate a large-scale reorganisation of neural resource allocation to prioritise salience network (over executive control network) connectivity during stress, facilitating rapid action and reorienting of attention (at the expense of higher-order cognition [60; for reviews see 35, 61]. Interestingly, a recent study among 355 participants found that those people who had more pronounced cortisol responses after stress, showed a stronger increase in connectivity within the salience network, but decreased default network coupling (both within-DMN connectivity, and between network connectivity)^12^. Finally, using graph analysis, Reinelt et al. ^13^ showed that stress increases the centrality of the thalamus, and that this increase persists for upwards of 105 min after the stressful event.

Thus far, little attention has been given to studying the effects of stress on time-varying or dynamic functional connectivity. While traditional static functional connectivity measures provide an estimate of network configuration averaged over a full duration of a scan run, dynamic functional connectivity (DFC) can identify moment-to-moment changes in network configuration in the order of several seconds^14^. Dynamic connectivity can capture trait-like, self-reported phenotypes as effectively as static functional connectivity, as well as having an advantage in capturing task-related metrics^15^. We thus reasoned that DFC may be particularly relevant in capturing a transient condition such as acute stress.

In prior work, we used DFC analysis to identify two connectivity states related to high and low arousal^16–18^. A high arousal state was characterized by high intra-network connectivity, particularly in control areas, and strong anti-correlation between multiple task-positive networks and the default mode network, while a low arousal state was characterized by lower intra and inter-network connectivity. These states fluctuated on a seconds-to-minutes timescale, and were related to eye closure and sustained attention after sleep deprivation^18^. Later studies showed that these states were systematically and consistently modulated by this manipulation. Indeed, we found that coupling between these states in a population of healthy undergraduates fluctuated in tandem according to whether one is well rested or sleep deprived^17^. Based on these findings, we reasoned that the increased arousal caused by stress would result in the reverse effect on functional connectivity than sleep deprivation: an increase in time spent in the high arousal state, and a decrease in time spent in the low arousal state.

Mindfulness is the second psychological focus of the current study. This concept was introduced as a tool in psychotherapy in the 1980s^19^, and is now an empirically supported method to mitigate the effects of chronic stress^20,21^. It has subsequently been discovered that even in the absence of training, individuals differ in their levels of dispositional mindfulness^22^, and this natural variation also predicts how one will respond to stress. For example, Brown et al.^23^ demonstrated that high self-reported mindfulness was associated with dampened affective and corti sol response to the TSST.

Researchers have also used resting-state functional connectivity to study the correlates of naturally varying trait mindfulness^24^ and the effects of mindfulness-based training^25,26^. While results from these experiments are mixed, there is some agreement that mindfulness is associated with increased connectivity within the default mode network (DMN), and in particular between the posterior cingulate cortex and ventromedial prefrontal cortex, greater anti-correlations between the DMN and attentional/control areas, and an increase in connectivity between regions of the ventral attentional network (particularly the insula) and executive control areas (see ^27^. Furthermore, studies have also found variation in dynamic connectivity that corresponds with trait mindfulness^27–29^. In a recent study, we identified a mindfulness-related connectivity state (which we named the "task-ready" state) that recapitulates some of the most robust features of findings from static connectivity; high levels of intra-network connectivity in the DMN and ventral attention network, and strong anti-correlations between these same networks^28^.

In spite of these intriguing inter-relationships, a unified study incorporating mindfulness, stress, and functional connectivity has not yet emerged, and in general, there has been a lack of experimental data bridging physiology, time-varying brain connectivity, and subjective report in any domain. To fill this knowledge gap, we designed an exploratory experiment to address the broad questions of i) how dynamic functional connectivity in resting-state fMRI changes following a stressor, ii) how trait mindfulness moderates this response, and iii) the nature of the relationship between trait mindfulness and arousal following a stress response. We tested several specific hypotheses to answer these questions. Our first prediction is that acute stress would lead to an increase in a “high arousal state” and a decrease in a “low arousal state”, in contrast to that observed from sleep deprivation^17^. We next predicted that trait mindfulness would correlate with the proportion of time spent in a third connectivity state, the task-ready state (which was associated with trait mindfulness in a previous study^28^), and that higher levels of this state at baseline would in turn be associated with a smaller stress response. Finally, our third prediction, primarily communicated in a separate report^30^, was that high trait mindfulness would be associated with smaller increases in self-reported stress and cortisol concentration in response to a stressor. Our data support this hypothesis. Relevant to this report, we focus on how this relationship between trait mindfulness and subjective stress modulates the incidences of the connectivity states.

## Results 

41 healthy individuals underwent a social evaluative stress induction (the TSST), after which they were scanned to measure their resting state connectivity changes due to stress (see Fig. 1). At various time points participants provided subjective ratings of stress and alertness, and also provided saliva samples for cortisol analysis. Results from the behavioural ratings and cortisol assays have been reported in a separate communication (^30^, see Supplementary Fig. 2). Relevant to this report, we found that increases in self-reported stress on the TSST and salivary cortisol were correlated with trait mindfulness (stress: r = − 0.41; cortisol AUCg: r = − 0.38). In contrast, alertness between pre-TSST and post-TSST did not show a significant increase.

### Dynamic functional connectivity states

Each participant completed one resting state scan prior to the stress induction (RS1), and two resting state scans immediately after stress induction (RS2, RS3). To obtain the dynamic connectivity time courses for these resting state runs, connectivity matrices across 114 cortical ROIs were calculated within 7 TR-wide sliding time windows. To identify distinct connectivity profiles (DFC states), the resulting connectivity matrices were fed into a k-means clustering analysis (k = 5). This number of clusters was chosen consistent with previous studies^52^; See Methods for details.

**Figure 2:**
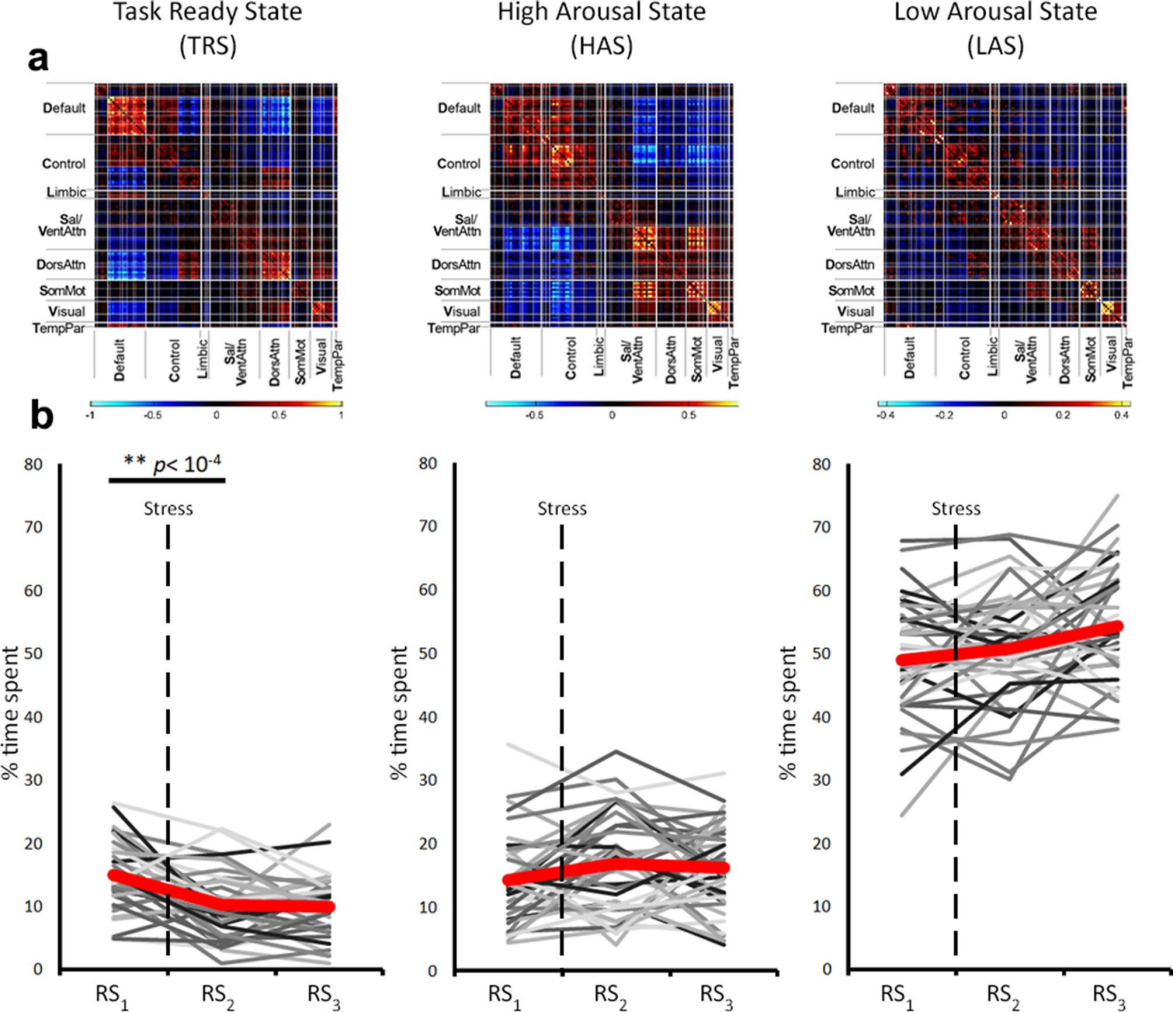
(**a**) Connectivity centroids of the task-ready state (TRS), high arousal state (HAS) and low arousal state (LAS). (**b**) Individual trajectories and grand means of changes in the states over the three resting-state scans. Significant differences were found between RS_1_ and RS_2_ for the TRS.

Three of the resulting connectivity states closely resembled the high arousal state and low arousal state, and task-ready state as described in our previous studies (^16–18,28^; Illustrated in Fig. 2). The high and low arousal states have been previously identified in the context of varying states of sleep deprivation^16,17^ and have been behaviourally associated with alertness/sustained attention performance^18^. The high arousal state is characterised by strong correlation between DMN and ECN, strong anti-correlation between these networks and the salience network, dorsal attention network, somatomotor network, and visual network, and strong within-network coupling for all networks. The low arousal state (LAS), on the other hand, is characterised by low inter and intra-network connectivity. The task-ready state has been identified in the context of individual differences in trait-mindfulness^28^. It is characterised by strong connectivity within the DMN, but anti-correlation between the DMN and other networks (particularly dorsal attention and executive control networks). The two remaining states have not been named previously, as no functional correlates have been identified.

To revalidate whether the 5-cluster solution as applied here matched the DFC states as identified in our previous studies^17,28^, Spearman correlations between the pairs of states were calculated, resulting in overall high concordance between centroid pairs (all rho > 0.82; See Supplementary Fig. 1a). While this suggests that the DFC state were reproducible across studies, there was not always an unambiguous one-to-one match (See Supplementary Information). Therefore, we further compared the current states to a dataset of N = 173 subjects, to which we applied the same analysis pipeline (See Supplementary Information for details), resulting again in high correlations (all rho > 0.92, see Supplementary Fig. 1b). The canonical centroids derived from the larger dataset are freely available as a resource on (https://github.com/awakelab/Dynamic-Functional-Connectivity-MTD).

### The effects of stress induction on DFC states

We performed pre-planned paired-samples t-tests on the three named states between RS_1_ (pre-stress) and RS_2_ and between RS_2_ and RS_3_ (both post-stress) to test whether any of these changed as a result of stress or recovery from stress respectively. Between RS_1_ and RS_2_ (i.e. as a result of performing the TSST), we observed numerical but non-significant increases in the LAS and HAS (Fig. 2b middle & right panels), and a significant reduction (t = 4.76, *p* < 0.001) in the TRS (Fig. 2b left panel). There were no significant changes in proportion of time spent in any of the states between RS_2_ and RS_3_.

### Dynamic connectivity changes correlate with changes in cortisol concentration, but not self-reported stress

To test our second exploratory hypothesis, we conducted bivariate correlations between the change in self-reported stress and alertness, and changes in the HAS (∆HAS) and LAS (∆LAS) between RS_1_ and RS_2_. None of these correlations were significant (all *p* > 0.05).

**Figure 3:**
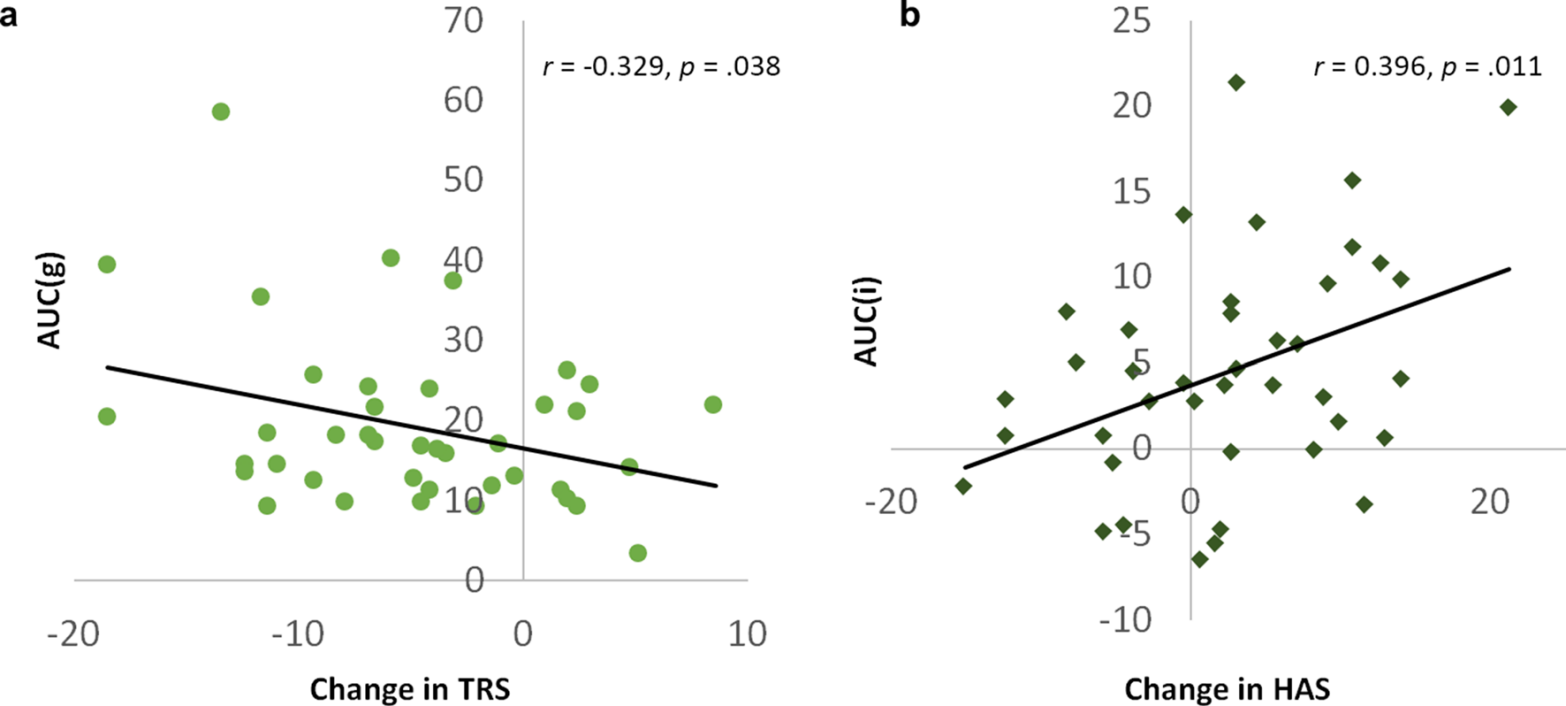
Correlations between cortisol concentration and the change in proportion of time spent in the task-ready state (TRS), high arousal state (HAS) and low arousal state (LAS). (**a**): total area under the cortisol curve (AUCg) is significantly correlated with ∆TRS; (** b**) area under the curve of cortisol increase due to stress (AUCi) is significantly correlated with ∆HAS.

We next tested for associations between changes in objective stress (cortisol) by correlating total cortisol (AUCg) and cortisol increase (AUCi) after RS_1_ and RS_2_ with ∆HAS, ∆LAS, as well as change in TRS (∆TRS) over that same period. We found a significant positive correlation between ∆HAS and cortisol AUCi (r = 0.39, *p* = 0.01) and a significant negative correlation between ∆TRS and cortisol AUCg (r = − 0.33, *p* = 0.04) (Fig. 3). However, these effects did not survive multiple comparisons correction over the six tests.

### Decrease in Task Ready State correlates with trait mindfulness

**Figure 4:**
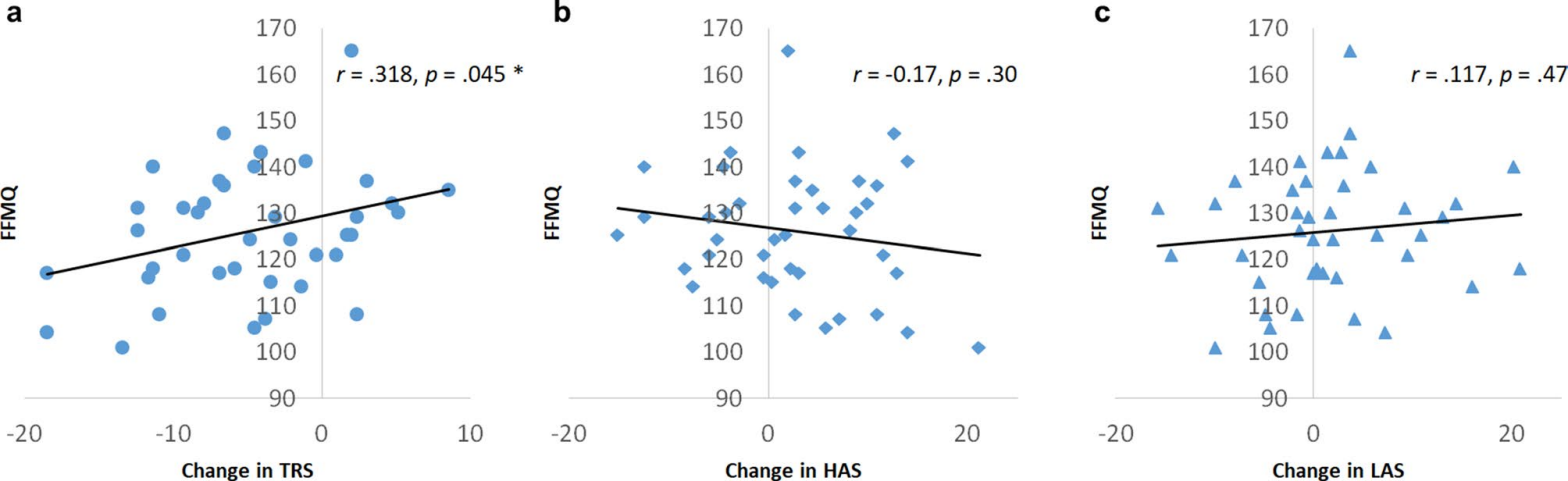
Trait mindfulness measured with the Five Facet Mindfulness Questionnaire (FFMQ) correlates positively with the change in proportion of time spent in the task ready state (TRS) due to stress (**a**), but is not associated with the arousal-related states (**b**,**c**).

Given our previous findings linking the TRS with trait mindfulness ^28^, we conducted further analysis to test if a similar relationship held in this dataset. Contrary to our a priori hypothesis, we did not find a correlation between trait mindfulness and TRS at baseline (i.e. in RS_1_) (r = − 0.24, p = 0.13). Instead, we found that trait mindfulness was significantly correlated with the ∆TRS (r = 0.32, p = 0.045), with greater trait mindfulness associated with a smaller decrease in time spent in this state. This relationship did not hold true for either of the arousal-related states (Fig. 4), and the effect did not survive multiple comparisons correction for these three tests.

### High and Low Arousal States show coupling in two datasets

**Figure 5:**
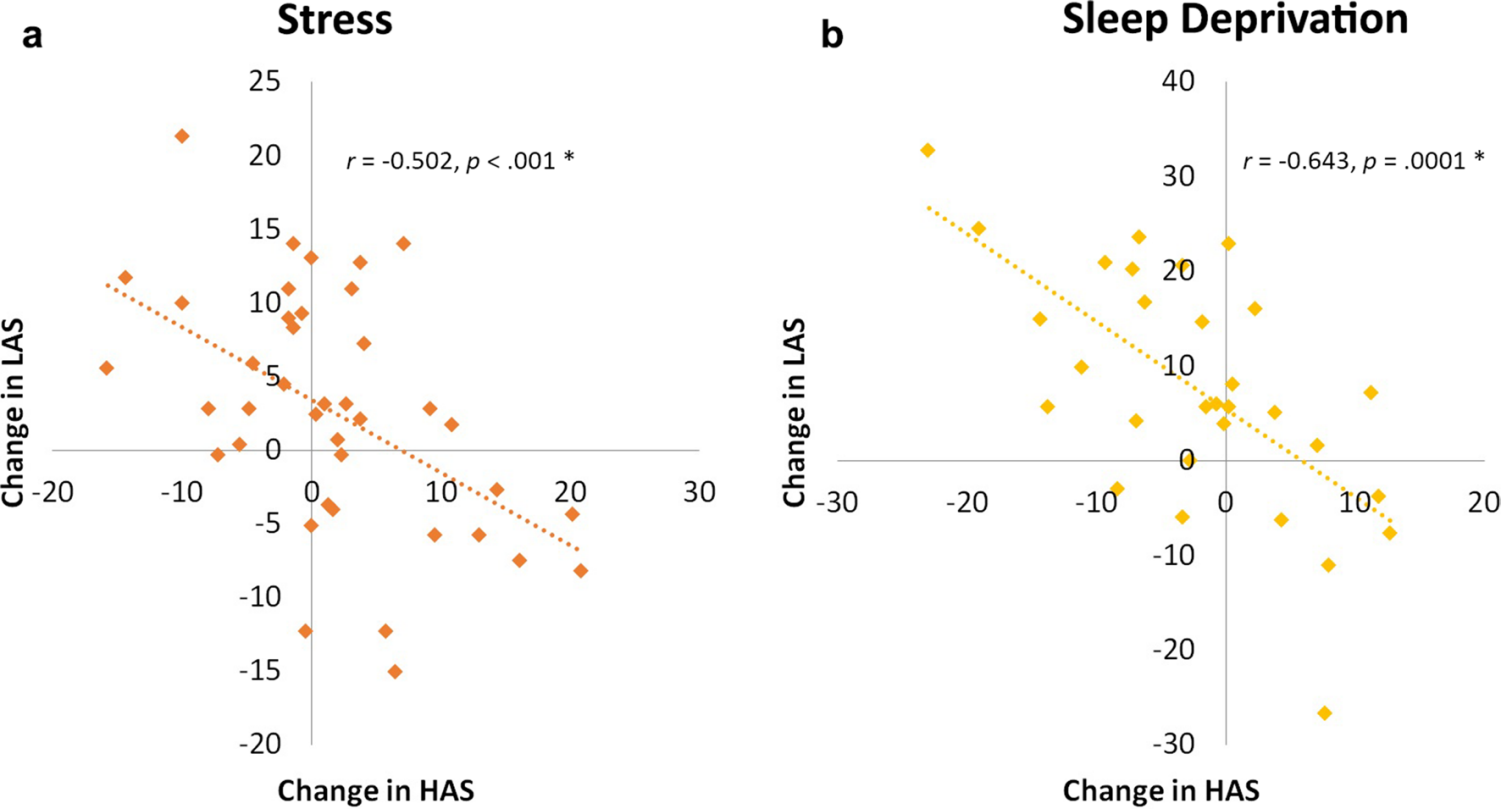
The high arousal state (HAS) and low arousal state (LAS) are coupled and change in tandem across state in two different datasets, (**a**) due to stress, and (**b**) as a result of 24 h of total sleep deprivation.

We tested for coupling among the three named states by correlating ∆HAS, ∆LAS, and ∆TRS between RS_1_ and RS_2_. Only one of these relationships was significant: we observed a negative relationship between ∆HAS and ∆LAS over this period (Fig. 5a; r = − 0.50, *p* =  < 001). For comparison, we performed a similar analysis on data from our previous report ^17^, a within-subjects design with fMRI scans obtained in subjects who had undergone 24 h of total sleep deprivation (compared with when they were well-rested). Again, ∆HAS and ∆LAS between the sleep-deprived and well-rested scans were tightly coupled (Fig. 5b; r = − 0.64, p < 0.001).

**Figure 6:**
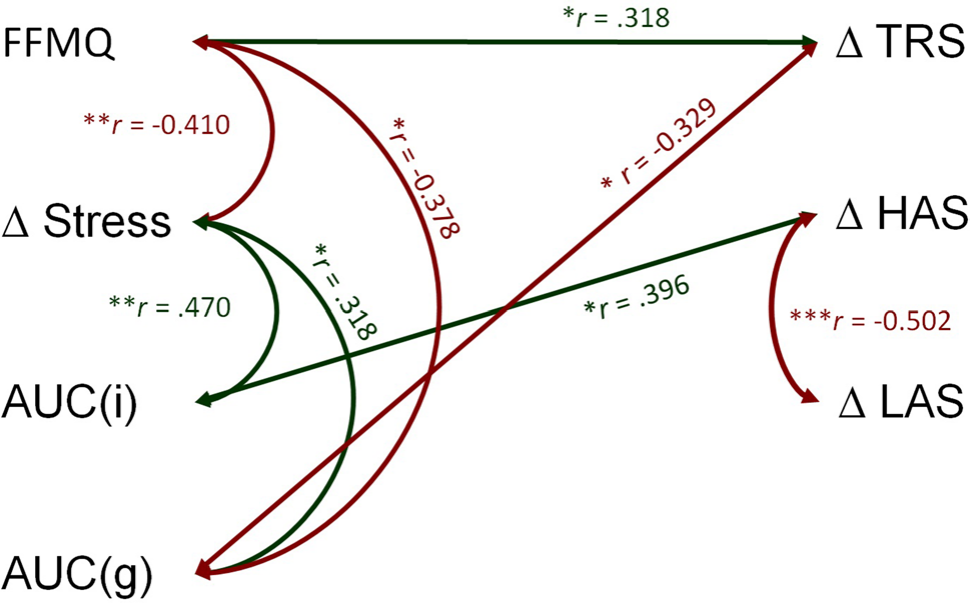
Significant relationships and correlation coefficients among variables of interest. FFMQ = Five facet mindfulness questionnaire, AUCi = increase in area under the curve (cortisol), AUCg = area under the curve with respect to ground (cortisol), TRS = task-ready state, HAS = high arousal state, LAS = low arousal state. *p < .05, **p < .01, *** *p* < .001.

To illustrate the interrelationship between self-reported data, cortisol, and dynamic connectivity states, we have summarised our findings in Fig. 6.

## Discussion

In the current study, we tested the effects of experimental stress induction on dynamic functional connectivity. Contrary to our hypotheses, no overall changes in high and low arousal DFC states (LAS/HAS) were found after stress induction, and no baseline correlation was found between trait mindfulness and a third DFC state previously associated with mindfulness (termed the "Task Ready State", TRS). Instead, inter-individual differences in the degree of DFC state changes after stress were associated with differences in cortisol response and trait mindfulness. These effects, however, were subtle (i.e. did not survive multiple comparison correction), and warrant replication in targeted future studies.

The first association found was a positive correlation between the change in High Arousal State (HAS) after stress and cortisol response. Despite finding no group-level change in HAS, individuals who displayed an increase in HAS after stress also showed stronger increases in cortisol (AUCi). This pattern may reflect that the effects of stress on the HAS connectivity state are subtle and may only show in those individuals who respond most strongly to stress.

Alternatively, it is possible that the high and low arousal state are related more directly to sustained attention than to arousal *per se*. Thus far, the strongest behavioural link to time-varying connectivity has been found in the context of sleep and wake transitions, and sustained attention performance during sleep deprivation^18,31–33^. While the ability to sustain attention is partially mediated by arousal, the two constructs are not synonymous; arousal is regulated via ascending noradrenergic projections from the brainstem to the thalamus, while sustained attention relies at least in part on top-down, thalamo-cortical circuits^34^. Given our pattern of findings, it is possible that the HAS and LAS index top-down readiness to respond, but not tonic, bottom-up arousal. In support of this possibility, we note that many of the strongly connected edges in the high arousal state centroid originate from the executive control network as well as areas in the dorsal attentional stream.

Another possible interpretation of the results is that increasing arousal via stress may not lie on the same dimension as dampening arousal using sleep deprivation. Indeed, there is evidence that subjective stress may manifest differently from physiological changes^35^. While noradrenergic activation changes in opposite directions during stress (increase) and sleep deprivation (decrease), cortisol is found to increase both after stress and after sleep deprivation^36^.

Relevant to this matter is that increases in noradrenergic activity during acute stress are directly related to the redistribution of resources from the executive control network to the salience network^12^. This is thought to promote prioritisation of rapid, bottom-up processing of threatening stimuli, over more controlled top-down processes ^37–39^. Noradrenergic activation however is short lived, and often returns to baseline quickly after the termination of a stress induction. Neuroimaging studies relating noradrenergic activity to functional connectivity changes often rely on connectivitymeasured during the stressful experience (e.g. threatening movie viewing^37^), while resting state scans acquired after the stress induction (as applied here) may be more suited to capture the slower cortisol response (e.g.^12^).

The second association found indicated that changes in a third dynamic connectivity state, the task ready state (TRS), were correlated with individual differences in trait mindfulness and overall cortisol output (AUCg). In previous work^28^, we reported that time spent in the TRS was correlated with trait mindfulness (in the absence of a stress manipulation)^40^. We did not replicate this baseline association in the current study. This belies our previous claim that the TRS encodes a constant, trait-like propensity to be mindful, and instead suggests that it is a functional configuration that is less vulnerable in mindful individuals during systemic challenge.

Other studies relating static^24^ or dynamic^27,28^ connectivity markers to trait mindfulness, or changes in functional connectivity following mindfulness training ^41^, have implicated the insula and default mode network as key nodes associated with mindfulness^42^. The distinguishing features of the TRS show good concordance with this extant literature, with strong within-network correlations in the default-mode network and salience network, and anti-correlations between the default mode network and key nodes of this network, including precentral gyrus, the anterior cingulate cortex and the insula. In our previous report, we posited that the TRS may play a role in supporting cognitive flexibility^28^, and this module is known to be impaired by stress, particularly in men^43^. While we did not measure cognitive flexibility in this paradigm, our current results provide additional impetus to formally test this hypothesis.

It should be emphasized that the found associations (both between HAS changes and cortisol, and between TRS changes and mindfulness/cortisol), were only significant before multiple comparison correction. While this is the first exploratory experiment to examine at the three-way relationship between changes in dynamic connectivity states, cortisol, and trait mindfulness, it remains imperative that these associations are verified in further work, and further research is needed to uncover their precise relationships.

Given the preliminary nature of the current results, several limitations need to be highlighted. First of all, it is useful to note that the neural signatures of stress-related brain connectivity are only partially mirrored in the identified DFC states. For instance, the HAS is characterised by high within and between-network connectivity of the salience network (which is also a hallmark of stress-induced network reorganisation^6,7,12,37^). However, the HAS is also characterised by strong anti-correlation between the DMN and salience network, which seems to be opposed to reported stress-related connectivity changes^3–5,8^. In addition, within-network connectivity in the DMN and executive control network is high in the HAS, while these are observed to be decreased during stress (more in line with the LAS). The TRS expresses some aspects that seem to directly oppose a stress-connectivity state (i.e. high within DMN coupling, and strong anti-correlation between DMN and salience network), however, the TRS was not hypothesized to be a direct reflection of stress-related arousal. As such, it seems likely that there is no simple one-to-one mapping of the identified dynamic connectivity states and connectivity changes commonly observed after stress. A relevant limitation of the current method is that it primarily captures cortical connectivity configurations. As the stress response strongly depends on the subcortical circuits including the amygdala and ascending noradrenergic projections originating from the brainstem, it is likely that cortical connectivity states do not cover the full spectrum of these effects.

A second limitation is that, in the light of the current findings, we note that our chosen labels for the identified DFC states (high arousal, low arousal, task-ready) may be slightly misleading as to their functional significance, notwithstanding that we have previously acknowledged that they are highly unlikely to represent one-to-one mappings onto psychological states^17^. While we have used them here for consistency with our prior work, we acknowledge that approaches to describing the connectome, such as fuzzy classification techniques involving principal component analysis (74), rather than assigning windows to discrete states, might offer clearer insights into precise brain connectomics. The findings also caution more generally against assigning labels to states prematurely (or perhaps altogether, in the light of the complexity of mapping connectomics to behaviour).

Furthermore, several limitations of the current study design must be highlighted. While we have attempted to control for several confounding variables (e.g. age, sex, diurnal cortisol variation) found in previous studies, we are limited by other factors. As mentioned in the Methods section, our sample was restricted to healthy young male participants of Asian ethnicity. This allowed us to study the targeted mindfulness-stress-brain connectivity associations within feasible sample size ranges, while keeping other factors stable. A clear downside of this approach is that it leads to the exclusion of female and non-Asian participants, and participants in different age ranges, which hampers the generalisability of our results in other populations. This is particularly important as both the mechanisms of the stress response^44^ may differ between males and females. Ideally, future studies should aim to further expand these findings and test the generalizability in more diverse samples.

Another limitation is that we did not include a non-stressed control group in this study, nor was sleep history regulated prior to the study. While incorporating a control condition could have strengthened our findings, it is unlikely that associations between mindfulness, cortisol reactivity and changes in connectivity states would be observed under conditions of no-stress. Studies that included a control condition have shown that a non-stressful control situation did not induce associations between mindfulness and cortisol reactivity^23^, while imaging studies have reported that changes in brain connectivity were primarily driven by the stressful condition^13^.

Lastly, it should be noted that the effects of stress on functional brain activation and connectivity may critically depend on the type of stress induction used, and the stage of stress procedure during which brain activation is sampled (see^39^ for a review). Studies using viewing or imagery of stressful content (e.g. aversive movie clips) have consistently found increases in salience network and DMN activity^12,45,46^, while other studies using cognitive tasks and negative social evaluation to induce stress, have found decreased activation of aspects of the salience network during the stress induction^47,48^, and mixed findings for the DMN^48–50^. Connectivity changes measured directly after stress induction (as was done in this study) have shown increased DMN-salience network coupling^4,5,7^, and increased connectivity between the salience network and other networks^9^. Sustained DMN-salience network coupling at longer time scales after stress induction (30 min or longer) has also been found^3–5^. The effects in the time period after stress induction are likely a result of temporally dynamic balance between rapid sympathetic adreno-medullary activation and slower cortisol responses. Different alterations in brain connectivity might still be found in context of chronic stress^51,52^ or stressful early-life events^53^.

In summary, we utilised time-varying connectivity to further explore the functional significance of three connectivity states that were first identified in previous work. We aimed to link these DFC states to stress reactivity and its modulation by trait mindfulness. While overall changes in DFC states after stress induction were not as hypothesized, inter-individual differences in the degree of change were related to cortisol response and trait mindfulness. These findings suggest a potential novel path via which mindfulness might moderate the stress response, and also hone our definition of how dynamic connectivity is altered by arousal and stress. Verification of these findings in future studies, however, is warranted.

## Materials and methods

### Participants

41 male participants were recruited from the National University of Singapore through online advertisements and word-of-mouth. We restricted our sample to healthy young (18–35y) male participants of Asian ethnicity, to arrive at a relatively homogeneous sample^30^. We chose this approach as it allowed us to study relatively subtle inter-individual variation in the variable of interest while keeping other known influences on the stress response stable^35,54^. Importantly, this approach allowed us to study these effects within feasible sample size requirements. Admittedly, this results in inclusion of a limited demographic, and generalizations beyond this demographic should be met with caution. One participant was excluded from the study for non-compliance to the protocol instructions, resulting in a final sample size of 40 (mean age (sd) = 23.03 (3.23)). All participants were screened to ensure that they had no history of long-term physical or psychological disorders, for right-handedness^55^, and for normal or corrected-to-normal vision. Participants were only admitted to the study if they had no prior knowledge of the study protocol, or exposure to similar social stress studies. Finally, they were excluded if they reported any contraindications for MRI scanning. The study was approved by the National University of Singapore Institutional Review Board. All participants provided written informed consent, and were reimbursed with SGD $50 for their participation. All methods were performed in accordance to the approved guidelines.

### Study protocol

All testing sessions were conducted between 14:00 pm and 17:00 pm to account for diurnal fluctuations of cortisol^56^. Participants first completed a 20-min computerized cognitive task (results not reported in this communication), followed by a 10-min eyes-open resting-state fMRI (RS_1_). Participants then performed the Trier Social Stress Test (TSST; see below) as a stress manipulation. Thereafter, participants underwent 2 additional resting-state fMRI scans (RS_2_ and RS_3_), interspaced with a 5-min high-resolution MPRAGE structural scan. After the last MRI scan, participants remained in the lab for a 30-min recovery period, before they were debriefed and reimbursed for their time.

Throughout the study, salivary cortisol and subjective stress ratings were collected at specific times during the protocol. The time points for all major data acquisition milestones were dictated by a pre-determined time schedule that research assistants adhered to as closely as possible; Fig. 1 shows this experimental protocol with timing relative to the start of the TSST, together with the actual acquisition timelines of each individual participant.

### Stress manipulation

The Trier Social Stress Test (TSST) is a common laboratory-administered protocol to induce psychosocial stress^1^. At the start of the test, a research assistant informed the participant that they had to prepare a speech for a mock job interview in front of two "behavioural experts". They were told that their performance would be video recorded and analysed. Participants were then left alone in a room for 5 min with writing material. They were allowed to make notes during this time, but these were collected from them at the end of the preparation period. Participants were then ushered by the research assistant to an adjacent room and introduced to the evaluation panel, which was always comprised of one male and one female confederate. Panellists were trained to withhold any social feedback and to maintain a neutral disposition at all times. We positioned a camera in plain sight of the participant that appeared to be operational, although no actual video recording was conducted. The participant was cued to begin their prepared speech, which was terminated after 5 min. If they fell silent or ran out of material before the end of the 5-min period, one of the panellists prompted the participant to continue by selecting an appropriate question from a predetermined list. In the second 5-min interview period, participants were then made to perform an unexpected mental arithmetic task. They were told to subtract 17 repeatedly from 2023 until they reach zero; to further induce stress, participants were urged to pick up their pace during long pauses, and had to restart their count if mistakes were made.

### Salivary cortisol

To assess stress reactivity, we collected salivary cortisol using Salivette (Sarstedt, Nümbrecht, Germany) at five time points (− 30, 0, + 20, + 60, + 90 min relative to stress onset; see Fig. 1). During each cortisol collection, participants were instructed to place the cotton swab in their mouth for 2 min and told to avoid contamination with their hands. All salivettes were stored at − 75 °C before analysis in a commercial biotechnology lab. Saliva samples were centrifuged at 20 °C (1000 × g, 2 min) and analyzed using salivary cortisol immunoassay (IBL International GMBH, Hamburg, Germany) with a sensitivity of 0.005 µg/dL. The intra and inter-assay coefficients of variation were all under 4%. The cortisol values were positively skewed and were thus log transformed for statistical analyses. We calculated two measures of cortisol output using the trapezoidal integration method by Pruessner et al.^57^, using all five cortisol samples across the baseline, stress, and recovery period. Area under the cortisol curve with respect to ground (AUCg) was defined as the total cortisol concentration over the duration of the experiment. Cortisol concentration increase related specifically to the stress induction (AUCi) was calculated by subtracting baseline production from AUCg. These metrics have been used as an integrative measure of cortisol output after stress (covering both ascending and descending aspects of the cortisol response) in the context of psychological differences in stress reactivity (e.g. mindfulness^58–60^), and neuroimaging^48,50^. One subject did not produce adequate saliva sample for the immunoassay, and was not included in cortisol analysis.

### Subjective mood ratings

Subjectively experienced mood was measured at six time points (Fig. 1), typically at the same time as cortisol collection. Participants indicated their immediate levels of Stress, Energy and Alertness on a nine-point Likert scale from 0 = *not all* to 9 = *extremely*. For the remainder of the manuscript, we focus analysis on Stress and Alertness at only three of these points (T2-4).

### Self-reported mindfulness

We measured dispositional mindfulness using the Five Facet Mindfulness Questionnaire (FFMQ)^61^. The 39-item inventory evaluates five distinct components of mindfulness: Observing, Describing, Non-Reactivity, Non-Judging, and Acting with Awareness. Items were rated from "1 = *never or very rarely true*" to "5 = *very often or always true*", and reversed scored for Non-Judging and Acting with Awareness subscales. The questionnaire showed good internal consistency with a Cronbach's alpha coefficient of 0.85, and as such, we used full-scale FFMQ scores for analysis.

### fMRI acquisition

Resting-state fMRI scans were collected on a 3-Tesla Siemens PrismaFit system (Siemens, Erlangen, Germany) using an interleaved gradient echo-planar imaging sequence (TR = 2000 ms, TE = 30 ms, flip angle = 90°, field-of-view = 192 × 192 mm, voxel size = 3 × 3 × 3 mm). 36 oblique axial slices were obtained, and 300 volumes (10 min) were collected for each scan. High-resolution structural images were collected using an MPRAGE sequence (TR = 2300 ms, TI = 900 ms, FA = 8°, voxel size = 1 × 1 × 1 mm, FOV = 256 × 240 mm, 192 slices).

Participants were instructed to remain still and keep their eyes open during the resting-state scan while not thinking about anything in particular. To ensure compliance, concurrent eye videos were acquired using an MR compatible camera (12 M-I eye-tracking camera; MRC Systems GmbH, Germany) placed over the right eye. Pre-recorded auditory reminders (e.g., "Open your eyes.") were delivered whenever participants closed their eyes for more than 10 s. This procedure was previously used^62^ to ensure that participants kept their eyes open during resting-state scans, as this can affect the results of connectivity analysis^63^.

### Resting-state fMRI analysis

Resting-state scans were preprocessed in accordance to the previously described procedure in Yeo et al.^62^, using a combination of FSL^64^, SPM (Wellcome Department of Cognitive Neurology, London, UK), and FreeSurfer^65^. Briefly, preprocessing steps involved (i) discarding the first four frames of each run, (ii) slice time correction, (iii) head-motion correction using rigid body translation and rotation parameters, (iv) functional and structural images were aligned using Boundary-Based Registration following FreeSurfer surface reconstruction. Whole brain, white matter and ventricular masks were then defined based on structural segmentation, then transformed to subject space. White matter segmentation was performed with 1-voxel erosion. (v) Linear trend removal was subsequently performed, with bandpass temporal filtering (0.009–0.08 Hz), and linear regression of spurious signal (head motion (3 regressors for translation and 3 for rotation), whole brain signal, white matter signal, ventricle signal, and their derivatives). (vi) Functional data of individual subjects were then projected onto MNI-152 space, downsampled to 2 mm voxels and then smoothed with a 6-mm full width half maximum kernel.

Global signal regression was carried out as a part of the preprocessing pipeline to remove potential nuisance components in the data^66^. Global signal power, or the standard deviation of the average percentage change in the signal time course of the whole brain ^67^, was subsequently calculated.

Head motion was calculated based on two measures: framewise displacement (FD) and variance of temporal derivative of time courses over voxels (DVARS)^68^. Volumes having FD > 0.2 mm or DVARS > 5% were marked as high motion. As we intended to perform dynamic functional connectivity analysis, motion scrubbing – or the removal of high motion volumes—was not conducted as this removal can have an impact on the temporal pattern of the underlying functional connectivity^68^. Instead, one volume before and two volumes after each high motion volume were also marked, and these frames were interpolated from surrounding data. No subject was excluded from the analysis for having more than 50% of total volumes marked as high motion (Framewise Displacement: RS1 FD Mean(sd) = 0.0496 (0.0434); RS2 FD Mean(sd) = 0.0445 (0.0409); RS3 FD Mean(sd) = 0.0532 (0.0528)).

### Dynamic functional connectivity analysis

Dynamic functional connectivity analysis was performed using the multiplication of temporal derivatives method described by Shine et al.^69^. 114 cortical ROIs were first extracted from the 17-network parcellation by Yeo et al.^70^. The coupling between each pairwise set of 114 ROIs was then estimated by multiplying the first-derivatives of the averaged BOLD time series. Connectivity at each time point was then estimated by computing a simple moving average of the multiplied temporal derivative time course using the recommended window size of 7 TRs, for a total of 292 coupling matrices per participant, each containing 6441 (114 × 113/2) unique coupling values.

Coupling matrices were than concatenated across the 40 participants for all three resting-state fMRI scans, and k-means clustering was performed to classify each matrix using Manhattan (cityblock) distance as the cost function. We used a k = 5 solution, for consistency with our previous reports, and as recent work using a large (N = 7,500) dataset of resting-state scans suggests that this is an optimal number of clusters^71^. To confirm that our centroids were consistent with those obtained from previous analyses reported by our group^16,28^, we performed Spearman’s correlations between the two sets of centroids. We then calculated the proportion of the run spent in each dynamic connectivity state.

### Statistical analysis

All statistical analyses were conducted on SPSS 25.0 for Windows (IBM Corp., Armonk, N.Y., USA), and statistical significance for all analysis was set at *α* = 0.05 (two-tailed). Change scores of our primary outcome variables are calculated by subtracting scores pre-TSST from post-TSST scores. Changes in proportion of time spent in dynamic connectivity states (∆HAS, ∆LAS, ∆TRS) across the three resting-state fMRI scans were similarly calculated (RS_2_-RS_1_, RS_3_-RS_2_).

Paired samples t-tests were computed for proportion of time spent in each connectivity state using pre-planned comparisons between RS_1_ (pre-stress) and RS_2_ and between RS_2_ and RS_3_ (both post-stress), as well as self-reported stress, and salivary cortisol before and after the TSST.

Pearson's correlation was performed between i) the change in self-reported arousal and stress, and ∆HAS and ∆LAS, ii) between change in cortisol concentration and change in the connectivity states, and iii) between trait mindfulness and the task-ready state. Spearman's correlation was performed to determine the similarity between dynamic connectivity states found in this study with previously found states.

## Supplementary Information

Supplementary Information.

## Data Availability

Data reported in this study are available in a public repository at https://osf.io/6kr34/.
