# Supplementary material for: Inter-relationships between changes in stress, mindfulness, and dynamic functional connectivity in response to a social stressor
Source: Sci Rep. 2022 Feb 14;12:2396. doi: 10.1038/s41598-022-06342-0 (PMC8844001; doi:10.1038/s41598-022-06342-0)
Supplement: Supplementary file 1 — Supplementary Information. [file 41598_2022_6342_MOESM1_ESM.docx]

**Inter-relationships between changes in stress, mindfulness, and dynamic functional connectivity in response to a social stressor**

James Teng, Stijn A.A. Massar, Julian Lim

Supplemental information

Reproducibility of dynamic connectivity states

In several previous reports, we have referred to three named dynamic connectivity states, the “high arousal state”, the “low arousal state”, and the “task-ready state” [9, 10, 11, 20]. We have claimed that the centroids for these states are reproducible when unsupervised (k-means) clustering is used in independent datasets. While the states are easily distinguishable on visual inspection, and show high correlations across datasets, inspection of the full correlation matrix between datasets reveals that there is not an optimized solution where each state has one and only one best-matched counterpart; for example, the state previously identified as the TRS in Lim et al. [20] is more highly correlated with the unnamed "State 5" in the current experiment than the state that is identifiable as "TRS" (see Supplementary Figure 1a).

To address this ambiguity, we attempted to create more stable estimates of the DCS centroids by increasing the amount of data used. Resting-state data from four separate datasets collected between 2015 and 2019 on a 3-Tesla Siemens PrismaFit system (Siemens, Erlangen, Germany) were compiled, consisting of 122 undergraduate participants (50 males; mean (sd) age = 22.8 (2.91)) who had undergone resting state scans of variable lengths (between 5.5 mins and 8.5 mins). Subjects from two of the datasets provided two independent resting state scans, resulting in a total of 173 scans entering the canonical analysis. Of these, 30 participants (15 males; mean age (sd) =23.00 (3.59)) had undergone one scan after rested wakefulness, and one scan following total sleep deprivation. Only the rested scans were included. The four separate datasets were preprocessed and analysed in an identical manner as described in Materials and Methods. Unsupervised k-means clustering was performed on the resulting datasets to obtain the 5 "canonical" centroids. Using these, we clearly show that across both the current dataset as well as that of Lim et al. [20], all ambiguous state pairings are resolved, and an optimal one-to-one match of states can be found (as can be seen on the diagonal of the matrix of Supplementary Figure 1d and e). Between Teng et al. [10], one ambiguous pairing (between the unnamed States 4 and 5) remains. This analysis adds weight to the notion that the states under study are unique and reproducible across different samples, and suggests that the canonical centroids may be useful as templates for comparison across DFC analyses using the Yeo et al. [50] parcellation.

We have made the connectivity matrices of these canonical centroids freely available on our github at (https://github.com/awakelab/Dynamic-Functional-Connectivity-MTD-)


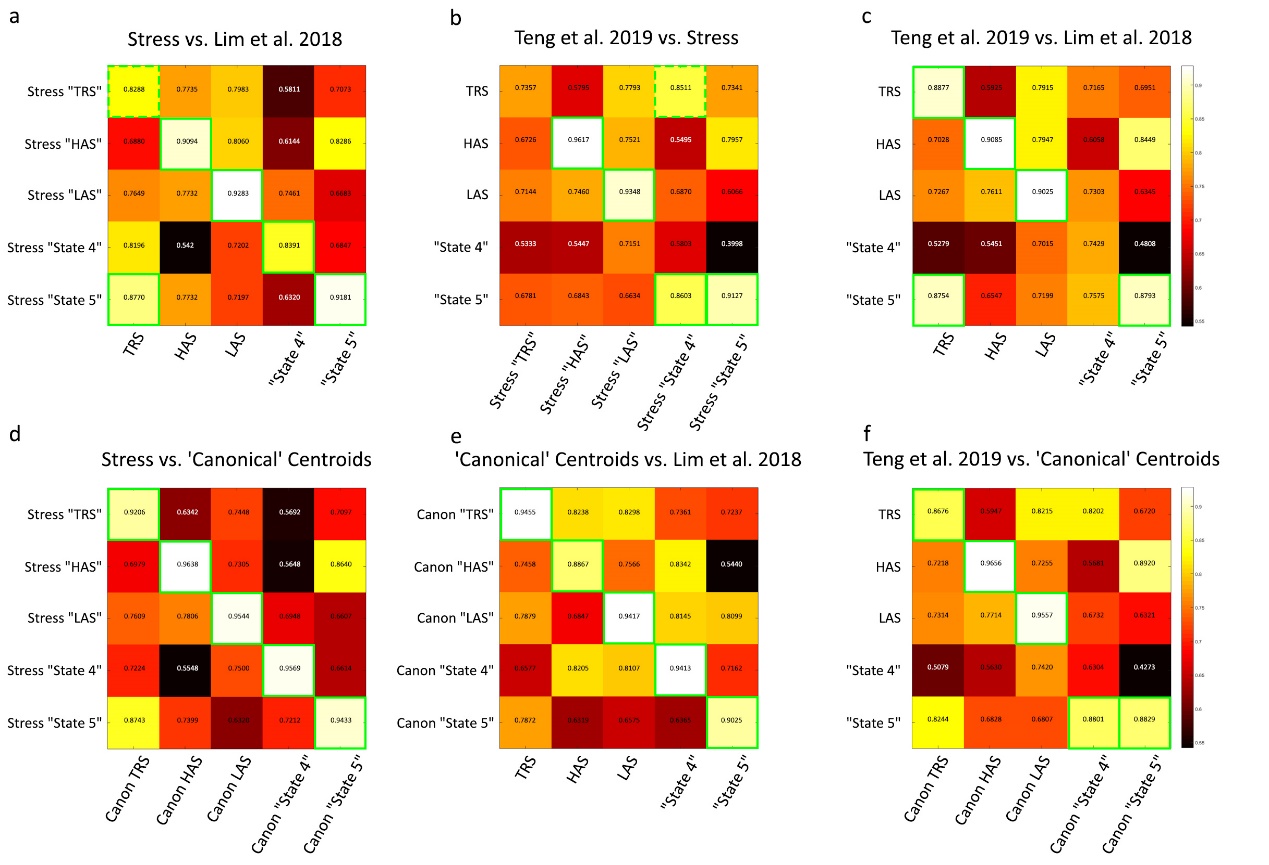


**Supplementary Figure 1**. Comparisons between centroids obtained from individual experiments, and between these datasets and the solution obtained clustering a larger (N =173) set of resting-state data. (a-c) Between the smaller datasets, some of the best-fit matches (shown in green squares) are found in the off-diagonals, indicating that there these have not been perfectly disambiguated (d-f) With the exception of States 4-5 in Teng et al., 2019, there is a clear one-to-one solution when compared with the canonical centroids.

Timecourse of cortisol and stress

As reported previously in a separated communication [22], salivary cortisol concentration and self-reported stress both increased significantly following the TSST


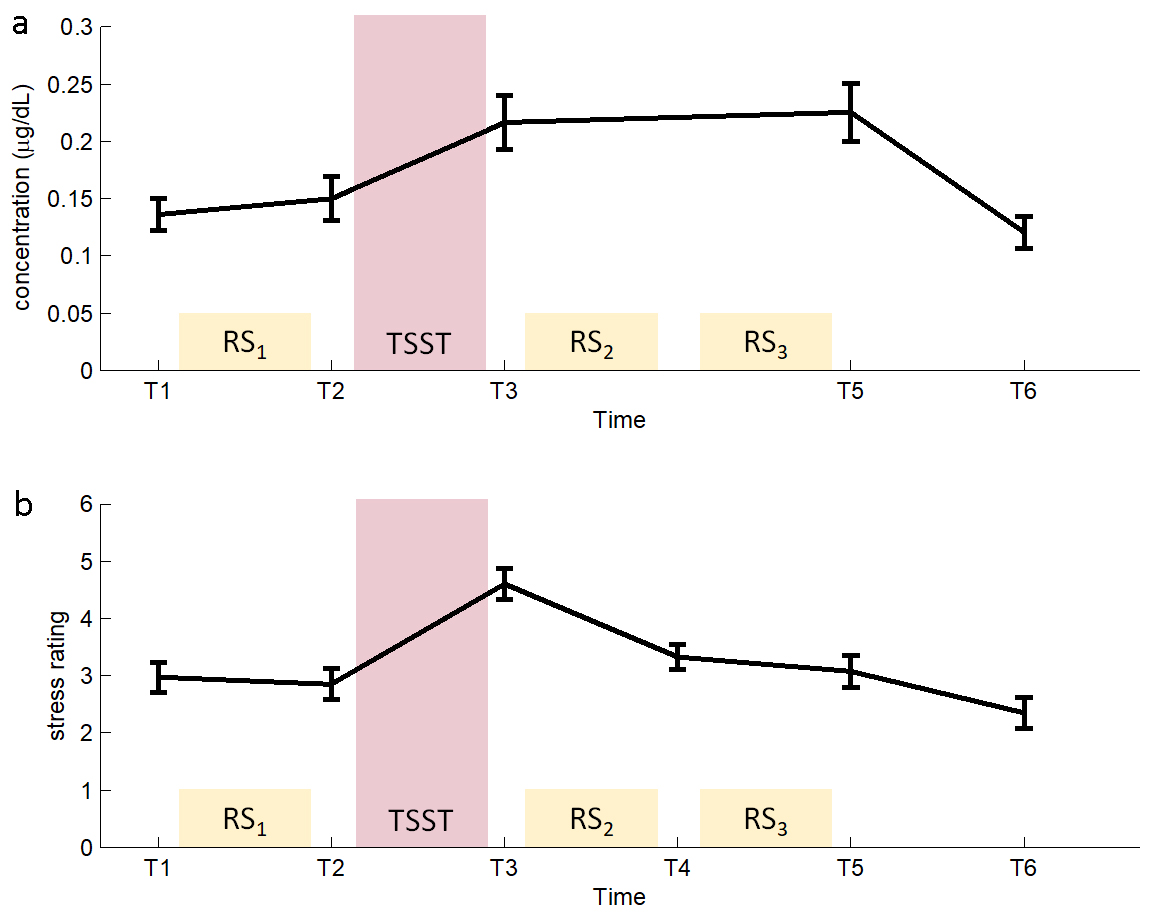


**Supplementary Figure 2.** Time courses of (a) cortisol concentration and (b) self-reported stress over the experimental protocol. Both variables increase significantly (p < .001) as a result of the TSST, with perceived stress showing a faster time course of recovery.
